# Extending alcohol brief advice into non-clinical community settings: a qualitative study of experiences and perceptions of delivery staff

**DOI:** 10.1186/s12913-018-3796-0

**Published:** 2019-01-07

**Authors:** Nicola Hall, John D Mooney, Zeibeda Sattar, Jonathan Ling

**Affiliations:** 1 Faculty of Health Sciences and Wellbeing, University of Sunderland, Sciences Complex, Sunderland, SR1 3SD UK England; 2 Faculty of Health and Life Sciences, Northumbria University, Newcastle, NE1 8ST UK England

**Keywords:** IBA, Brief intervention, Alcohol, Community, Qualitative, Perceptions, Experiences, Acceptability

## Abstract

**Background:**

At a population level, the majority of alcohol-related harm is attributable to drinkers whose consumption exceeds recommended drinking levels, rather than those with severe alcohol dependency. Identification and Brief Advice (IBA) interventions offer a cost-effective approach for reducing this harm. Traditionally, IBA interventions have been delivered in healthcare settings and therefore contextual influences on their use in non-clinical settings are not well understood.

**Methods:**

Qualitative face-to-face and telephone interviews with staff responsible for delivering a pilot IBA intervention across community settings in the UK. Interviews were recorded and transcribed verbatim. Inductive thematic analysis was used to identify key issues and the constant comparison method was employed to compare barriers and facilitators to implementation across and within settings.

**Results:**

A number of facilitators and barriers to delivery and implementation was identified across settings. These included familiarity with the customer base, working within public spaces, and assimilation of the intervention within existing role boundaries. Despite underlying concerns relating to the sensitive nature of the topic, most delivery staff felt their respective settings were appropriate for the delivery of the intervention and had proactively engaged members of the public with varying levels of risky drinking and readiness for behaviour change. Perceptions of actual or potential intervention success were conceptualised in relation to existing day-to-day role boundaries and responsibilities and the contexts in which they took place.

**Conclusions:**

Findings support the potential value of multi-setting community approaches to facilitate more inclusive engagement with IBA. By comparing experiences and views from staff responsible for delivering the intervention across different community settings, our findings provide insight into how intervention acceptability and success are framed across settings, and how the intervention is assimilated within everyday practice and role boundaries. This study also highlights key areas to be addressed when implementing IBAs in non-clinical community settings by staff with diverse levels of health-related knowledge, skills and support needs. Although essential, the need for adaptable training and delivery approaches across different setting types is likely to result in methodological challenges that need to be addressed when evaluating future interventions and setting-specific influences on behaviour change and health outcomes.

## Background

The health, social and economic consequences of excessive alcohol consumption on individuals, families, and communities are wide reaching [1]. At a population level, the majority of alcohol-related physical, psychological and social harm is attributable to excessive or hazardous drinkers whose consumption exceeds recommended drinking levels, rather than the smaller proportion of drinkers with severe alcohol dependency problems [2]. A preventive approach to alcohol problems is therefore advocated within a number of national and international programmes and policies to reduce alcohol-related harm [3, 4].

Evidence supports the use of individual brief advice and counselling interventions as a cost-effective means of influencing alcohol consumption [2, 5]. Although the content of alcohol brief interventions (ABI) varies between studies, core features tend to be that they are delivered by generalist healthcare workers, they target a population of excessive (or hazardous) drinkers that are not necessarily seeking help for alcohol problems and they aim to reduce consumption and related harm [2]. Identification and Brief Advice (IBA) is a form of ABI defined as a short face-to-face conversation about alcohol consumption at which a validated screening tool is administered to detect those drinking at hazardous or harmful levels [6]. IBA does not normally include interventions that involve more intensive or skilled counselling interventions that can be part of ABI. In practice however, the acronyms IBA, ABI and SBI (Screening and Brief Intervention) are often used interchangeably, with little consensus as to the differences between them. IBA tends to be the preferred term in the UK [7]. In the US, Screening, Brief Intervention and Referral to Treatment (SBIRT) programs have been implemented mainly within medical care settings to help identify patients at risk of substance abuse and dependence and refer those in need to appropriate treatment [8].

Primary care has been widely promoted as a key setting for the delivery of IBA. More recently, however, some large pragmatic trials have been unable to confirm previous claims for its effectiveness in this setting [9–11] . Questions have also been raised in relation to methodological limitations of the research evidence supporting the effectiveness of IBA interventions, their implementation and use in everyday practice, and their applicability to wider populations [12, 13]. Furthermore, the uptake of IBA within general practice remains low despite the introduction of financial incentives [13, 14].

In the UK, health policy support for the increased opportunistic implementation of IBAs in primary care has also been extended into secondary care and other health care settings [4]. Implementation of IBAs in alternative community settings, including social care, criminal justice and the community and voluntary sectors are also becoming more common [15–20] and is supported by national guidelines There has been considerable interest in community pharmacy, in particular, as an appropriate setting for the delivery of IBA in light of community pharmacists’ increasing role in public health and health promotion as well as their accessibility, particularly in areas of high deprivation [21, 22]. There is growing evidence to support the feasibility and acceptability of this setting for the delivery of IBA from both public and practitioner perspectives [23–25]. Identified barriers to implementation include public concerns about confidentiality and privacy [26, 27], and organizational obstacles, such as concerns about alienating customers, the pharmacy environment and lack of time, staff confidence and training [23, 27, 28]. Evidence to support the efficacy and effectiveness of IBA within community pharmacy and other community settings however is limited [29–31]. There is also little understanding of the mechanisms of impact and contextual factors that might support the widening of IBA delivery to non-clinical settings in line with a health in all policy approach [32] and, in the UK setting, the Making Every Contact Count agenda [33].

This paper reports on the perceptions and experiences of staff and community volunteers responsible for delivering a pilot IBA intervention across community pharmacy and other novel community settings, community health organisations and stores from a national supermarket chain, in order to explore the contextual influences on the delivery and implementation of IBA in non-clinical settings.

## Methods

This qualitative study was part of a wider mixed methods process evaluation of a pilot IBA intervention delivered across three community-based settings: community pharmacy, community health organisations, and stores from a national supermarket chain. The evaluation design was informed by Medical Research Council (MRC) guidance for the process evaluation of complex interventions (34). Qualitative semi-structured face-to-face and telephone interviews were completed with staff responsible for the delivery of the intervention across all three settings. The main research aim was to identify the key contextual influences on perceived appropriateness and feasibility of delivering IBA in alternative community settings by non-specialist staff. Interviews focused primarily on the exploration of contextual influences on implementation, intervention fidelity and perceptions of success. Fully exploring key issues at this feasibility stage was intended to allow identification of required changes to the intervention components or implementation structures before subsequent effectiveness evaluation in line with the MRC framework for the design of complex interventions [34].

### The pilot IBA intervention

The intervention was designed and piloted by the Drinkaware trust. This organisation is an independent UK-wide alcohol education charity, funded by alcohol producers and retailers to help reduce alcohol-related harm [35]. The target audience, midlife men drinking routinely at home, was chosen based on previous drinker segmentation analysis identifying a higher risk drinking segment of ‘Risky Career Drinkers’ within this population in the UK [36]. All materials were specifically designed with this audience in mind and the language used, style, products and visuals were targeted to optimise engagement with this group in a way that was non-stigmatising and engaging. Delivery was not intended to be exclusive to this group and the materials were designed to appeal to a broad audience. Promotional materials focused on a £50 prize draw incentive were designed to support initial proactive engagement with the public.

The screening element of the intervention consisted of the short form Alcohol Use Disorders Identification Test (AUDIT-C) [37, 38]. This has been validated for use in primary care populations and recommended for use in other settings, including community pharmacy [27]. It was adapted for use as a self-completion scratch-card. A “personal score” information leaflet was developed that was tailored to each risk category identified from the AUDIT-C responses and covered information on alcohol related-harm, prompts to motivate behavior change, and possible strategies for reducing alcohol consumption. This leaflet was used as a prompt to engage in a targeted brief conversation about alcohol consumption and handed to participants to take home with them. In line with good practice for improving the effectiveness of social marketing campaigns [39], embedding face to face discussion within an existing UK based health education campaign provided the opportunity for reinforcing an existing message with a different medium.

In the UK, there has been an increasing emphasis on the delivery of health promotion within Healthy Living Pharmacies [40] and community pharmacy was identified as an appropriate setting for the pilot. Organisations providing community health services and stores from a national supermarket chain were selected as comparator settings. These were chosen to allow exploration of contextual influences on the delivery and implementation in settings in which there is currently little evidence.

The approach to both training and delivery was mainly experiential in focus. All settings were provided with IBA “kits” that included all the information and resources required to deliver the intervention and were developed specifically to be self-explanatory and require minimal training or explanation for non-expert staff across settings. Staff responsible for delivering the intervention were encouraged to open discussions about alcohol consumption with members of the public scoring in the “increasing risk” category using the following 3 questions: How does your score make you feel?; What other benefits might you get from drinking a little less?; How do you think you could drink a little less? Staff delivering the intervention were asked to recommend to participants in the “high risk” category that they contact their GP or local specialist alcohol support services.

### Settings

The intervention was delivered across three different types of non-clinical community settings between November 2016 and February 2017. These consisted of: 14 community pharmacies in the North West of England; two community health organisations (one based in the North West and one based in South Central England) and 98 stores from the same supermarket chain across England, Scotland, Northern Ireland and Wales. The intervention was implemented flexibly across the setting types to fit in with existing services and ways of working. Delivery in the supermarkets took place over one day only (the same day in all 98 stores). Community pharmacies displayed promotional materials throughout the intervention period. Community health organisations delivered the intervention throughout the intervention period, but only on a “sessional” basis. This took place within different community sites, including shopping centres, cafes and city centre streets. These were selected mainly within areas associated with high levels of socio-economic deprivation. Overall, the intervention was delivered to 3559 members of the public. Returned Audit-C scratch cards demonstrated that staff across all settings had reached individuals from all three alcohol consumption risk categories (no/lower, increasing and higher) and age ranges.

### Participants and sampling

Semi-structured qualitative interviews (*n* = 31) were completed with staff responsible for delivering the intervention. These included: pharmacist and non-pharmacist staff in the community pharmacies; expert health and well-being advisors and lay volunteers in the community health organisations; and “brand ambassadors” employed by a third-party marketing organisation to deliver the intervention in the supermarket stores. Interviewee sampling and recruitment varied across settings. Staff from the supermarket setting were selected at random from a list provided by their employer and staff from community pharmacies were purposively selected to ensure variation of pharmacies in terms of geographical location and pharmacy size. Community health staff were purposively selected to provide accounts from staff who had delivered the intervention over a range of intervention sites.

### Data collection

Face-to-face semi-structured interviews were completed in all settings apart from the supermarket setting where, due to the large geographical spread of the interviewees, telephone interviews were conducted instead. A topic guide was developed in line with the aims of the wider evaluation, interviews covered perceptions and experiences of training and intervention delivery, perceived public engagement and reach, perceived appropriateness of the setting and other contextual influences on intervention delivery and success. The interview guide was flexible to allow opportunities to pursue participant-let topics. Interviews lasted between 20 and 60 min. All interviews were recorded, transcribed verbatim and anonymised.

### Data analysis

An inductive thematic approach to the analysis of qualitative data was employed [41]. QSR International’s NVivo version 11 Software was used to support data coding. Emphasis was placed on the identification of common and divergent themes across and within settings. Data analysis occurred iteratively and included: reading and re-reading the transcripts; generation of initial themes; reviewing and refining themes; and identification of patterns across and within cases and settings using the constant comparative method [42]. Results are reported using narrative description and data extracts identified by setting and the professional role of participants. Initial data coding was completed independently by NH and ZS with a high level of agreement. Consensus over final themes was reached through discussion.

## Results

A summary of the qualitative data collected within each setting, detailing the professional roles of participants, is provided in Table 1. Findings are reported in relation to a) perceived facilitators and barriers to intervention delivery and implementation and b) beliefs about intervention success.

**Table 1:** Summary of qualitative data collection

Setting	Qualitative Interviews
Community pharmacy	Face to face interviews
	6 pharmacists
	1 pharmacy technician
	2 counter staff
	2 health Champion/smoking cessation advisors
	1 pharmacy supervisor
Supermarket	Telephone interviews
	12 supermarket delivery staff
Community Health Organisations Total	Face to face interviews*
	2 smoking cessation advisors
	2 health and well-being advisors
	3 volunteers/team members
	**31 Interviews**

*Staff interviewed were able to reflect on experiences of intervention delivery across a wide range of locations and settings including: supermarkets, shopping centres, GP practices, mobile units, cafes, bingo halls, colleges, health centres and community gateways

### Perceived facilitators and barriers to intervention delivery and implementation

Table 2 summarises key barriers and facilitators to intervention delivery and implementation by setting. The sub-themes from this table also provide a useful framework for understanding underlying beliefs about setting appropriateness.

**Table 2:** Facilitators and Barriers to Delivery and Implementation

Sub-themes	Setting Specific		
	Supermarket	Community pharmacy	Community Health (range of site types)
Role legitimacy	*- Role legitimacy less clear*	*+ Fits well with changing responsibilities of pharmacy and contractual arrangements*	+Integration with other services (e.g. smoking cessation)
			+Strong role legitimacy
	*+ Compatibility with raising awareness and marketing approaches*		-Variability of roles
		*+Integrates with other services (*e.g. *MUR)*	
		*+Trusted health professional status and strong role legitimacy for pharmacists and healthy living champions*	
		*-Role legitimacy for other pharmacy staff more variable*	
Audience reach and engagement	*+ Wide audience reach*	*+ Time available when waiting for prescriptions*	*+Flexibility of multiple sites and wide audience reach in targeted hard to reach areas*
	*+ Information taken home for others*	*-High percentage of regular/repeat customers limits audience reach*	
		*-Variable reach of customer base and engagement*	*+Able to attend to wider range of social factors*
			*+Some sites (*e.g. *café) more time for discussion*
Level of information and materials	*+ Appropriate training and materials for setting*	*+Materials encourage permission to ask for advice*	*+ Materials attract attention*
	*+Displays, free giveaways and materials attract attention*	*-Additional “props” felt necessary*	*- Lack of flexibility to incorporate more creative local solutions*
	*- Perceived insufficient training and knowledge can result in lack of confidence*		
Dealing with a sensitive topic within public spaces	*- Not wanting to offend or embarrass*	*+Availability of private consultation room*	*+Support from wellbeing advisors available*
	*-Public space - not engaging those most in need*	*+Availability of support from trained health professional for counter staff*	*+Trained in provision of healthy lifestyle advice*
	*-Public space - not appropriate for dealing with sensitive*	*-Not wanting to offend or embarrass*	*- Public space - not engaging those most in need*
	*topics or people upset*		
		*- Public space – not engaging those most in need*	
		*-Lack of privacy at counter*	
Familiarity with customer base	*+ Existing relationships know customers well*	*+ Existing relationships – know customers well*	+Know customers well (when integrated with other services)
	*- Public expecting food testing/giveaways*	*-Familiarity affects engagement when “private” topic*	- Lack of time to develop trust
	*-Familiarity affects engagement when “private” topic*	*+Ability to follow progress of return customers*	
Dealing with a sensitive topic within public spaces	*- Not wanting to offend or embarrass*	*+Availability of private consultation room + Availability of support from trained health professional for counter staff*	*+Support from wellbeing advisors available*
	*-Public space - not engaging those most in need*		*+Trained in provision of healthy lifestyle advice*
	*- Public space not appropriate for dealing with sensitive topics or people upset*	*-Not wanting to offend or embarrass*	*- Public space - not engaging those most in need*
		*- Public space – not engaging those most in need*	
		*-Lack of privacy at counter*	
Physical spaces and environmental context	*+ Cue to action – proximity to alcohol sales*	*+ seen as health-related space*	*+Flexibility and variability of delivery “sites”*
	*- Conflict of interest – proximity to alcohol sales*	*-“busyness” of pharmacy setting*	*-External conditions in some sites (*e.g. *weather, noise)*
Organisational context	*+Dedicated staff time*	*+Existing links to other referral networks and services*	*+Volunteer training*
	*-Working on own with no support*	*-Lack of dedicated staff time*	*+Links to local knowledge and other referral networks*
	*-No existing links to or knowledge of other services*	*-Can conflict with other pharmacy priorities*	*+Dedicated staff time*
		*- Health professional status can limit honest responses*	*-Variable organisational support and priorities*

Key: + = facilitator/advantage; − = barrier/disadvantage

Most participants reported that their setting was appropriate for engaging members of the public in discussions about alcohol, although they legitimized these beliefs in different ways. Within community pharmacy, setting appropriateness was usually framed in relation to the extension of the pharmacy role, national contractual requirements to promote healthy lifestyles, and their participation in other locally commissioned alcohol awareness raising events or services.

*“Healthy living its part of what we have to do as part of our contract anyway and as well as being a healthy living pharmacy, it’s an additional requirement now.” (Community Pharmacy 2, Pharmacist)*

Supermarket and community health settings were felt to be particularly appropriate for reaching a wider audience base and members of the public who would not usually engage with clinical or health services. Community health staff, in particular, felt that a benefit of their setting was their flexibility to complete the intervention in a range of different sites. This allowed them to approach and reach different target groups, including those from areas of high levels of socio-demographic deprivation who traditionally have less contact with other services.

*"One thing beneficial is that we go into places less corporate or clinical. Really positive and beneficial things for us, grab people who don’t necessarily go in to speak to people [health professionals]." (Community Health Team, UI-1, Supervisor)*

There was recognition, nevertheless, that the nature and level of the intervention and materials needed to be setting appropriate and in keeping with the public nature of the spaces in which the intervention was being delivered.

*“The information we were given was appropriate at the level of talking to people in supermarkets. I think if you were a health care professional talking to somebody in private obviously you would need far more detailed information.” (Supermarket 3, Brand Ambassador)*

The intervention was delivered in public spaces across all settings and although this did not influence overall perceptions of setting-suitability, it was acknowledged that the lack of privacy may have influenced interest and engagement, under-reporting and the depth or impact of discussions. Some participants felt this may have been a barrier particularly for those who were aware of their need to reduce their alcohol consumption. The availability of a private consultation space within community pharmacies was seen as an important advantage within this setting. In practice, however, this was very rarely used unless the intervention was already integrated within existing services, such as Medicines Use Reviews (MURs) or smoking cessation services.

*“I just always bring it up anyway in when we are doing the smoking [cessation] and I think they’re a bit more honest … but when you’re outside in the shop we just sort of, I think they get a bit more embarrassed about it.” (Community Pharmacy 5, Counter Assistant/Smoking Cessation Advisor)*

Familiarity and existing relationships with the customer base were described as both facilitators and barriers to delivery depending on the context and beliefs about public perceptions of professional role boundaries.

*“It’s difficult to tell people they’re at risk and they should go and see a doctor you know, it’s like who are you to tell me?” (Supermarket 12, Brand ambassador)*

Some pharmacists reported that their status as “trusted health professionals” helped to engage customers, however, most encounters within this setting were completed by busy pharmacy counter staff who often did not have time to facilitate participation. One pharmacist, however, acknowledged that their role as health professional might make it more difficult for customers to be honest about their drinking consumption.

*“I think maybe because we are sort of in charge of their medicines and their health maybe they feel they didn’t want to be totally honest” (Community Pharmacy 4, Pharmacist)*

Interviewees from all settings reported feeling comfortable in engaging with the public, however some accounts reflected underlying anxiety due to the potentially sensitive nature of the topic and concerns about being seen to be “lecturing” about a behaviour usually couched in moral and negative terms, particularly when dealing with regular customers/clients. Experience of intervention delivery was reported to have increased confidence in engaging members of the public in discussions about alcohol across all settings, including the supermarkets where the intervention was delivered over one day only. Nevertheless, one Brand Ambassador described his concern and perceived lack of preparation when dealing with a customer who had scored in the high risk category*.* These experiences were rare, but highlighted that delivery staff in this setting were mainly working on their own with no access via their employing organisation to onsite support or established links to local support organisations or alcohol services.

Differences across and within settings in organisational structures, leadership, facilitation, resource and funding arrangements resulted in considerable heterogeneity in organisational and staff engagement. One pharmacist, for example, explained how their pharmacy had modified the intervention approach to accommodate a lack of resources:

*“while you’re waiting for us to find your prescription would you be able to help us out and fill in one of these scratch cards and here’s a leaflet as well”, rather than: “What score did you get, let’s go through this”, when it can become a bit invasive … [There’s] also a time aspect, … we haven’t got the actual manpower [for the recommended intervention approach] … (Community Pharmacy-3, Pharmacist)*

Other barriers and facilitators to delivery relating to the physical spaces in which each setting were delivered. Within the supermarket setting, for example, the proximity of intervention delivery to alcohol sales was seen on the one hand as an appropriate cue to action, but on the other, as a potentially problematic barrier to engagement.

*“…plus the fact that people purchase alcohol in the supermarket so I actually thought it was an appropriate place to have a discussion about alcohol.” (Supermarket 3, Brand Ambassador)*

*“I thought it was a bit awkward because obviously I was set up by the beer, wines and spirit aisle so whether that was the right place ... because it’s a bit too close to home but I’m not too sure it made too much difference to customers” (Supermarket 1, Brand Ambassador)*

**Beliefs about intervention success**

Evidence was provided from all settings that a range of people had engaged with the intervention with variation in levels of: alcohol consumption risk; awareness of the impact of their consumption levels; willingness to engage with the materials; and interest in changing their behavior. All settings gave examples of positive comments relating to members of the public engaging with the materials and reporting intention to make changes to their drinking habits. For some participants, these beliefs also affected their expectations about the success of the intervention in terms of facilitating behaviour change.

*“We’re giving them the information but I don’t think we’re giving them the tools to implement the information... If we had more training then perhaps we could take it further” (Community Pharmacy 1, Pharmacist)*

Familiarity with regular pharmacy customers facilitated awareness of reported changes to drinking patterns and allowed staff from this setting to also monitor and reflect on the successful impact of intervention engagement on drinking behaviour.

The community health setting was the most heterogeneous in terms of roles, perceived responsibilities and site types. For example, this setting included smoking cessation advisors who were trained in supporting behavior change and were delivering the IBA within the context of other services, experienced community health and wellbeing advisors and less experienced volunteers. These all delivered the intervention across different site types, including cafes, health centres, high streets and shopping centres with some focusing more on raising awareness and others on supporting behavior change or engaging with traditionally hard to reach groups. This meant that the intervention success was assimilated within every day practice in different ways, focusing on different elements of motivation and behavior change. The range of roles and potential overlap with existing responsibilities highlighted the need for clear intervention boundaries.

*“I said to her promise me you’ll go and see a doctor and then it was almost like I sort of ended the conversation because I’m not a counsellor, but I did what I was supposed to do” (Community Health 2, Smoking Cessation Advisor)*

**Fig. 1:**
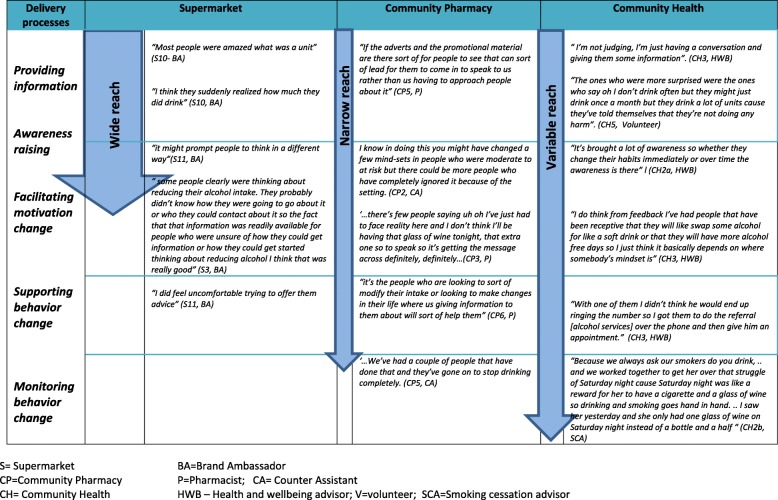
A conceptual model of reach, engagement and assimilation of the intervention within perceived role boundaries

A conceptual model, including data extracts, is provided in Fig. 1 to illustrate how reach, engagement and the assimilation of the intervention with existing role boundaries differed across settings. Arrows next to each setting illustrate where participants from each setting most often positioned their framing of intervention success and the extent of their expected intervention reach.

## Discussion

Our exploration of the perceptions and experiences of staff and community volunteers responsible for delivering IBAs within community pharmacy and other community settings provides novel insight into the contextual influences on the delivery and implementation of IBAs in non-clinical settings, as well as the ways in which intervention appropriateness and success are conceptualised across settings. We present a model that summarises and illustrates how reach, engagement and the assimilation of the intervention with existing role boundaries differ across settings.

Current guidelines and policy [4, 43] have encouraged the extension of IBA from primary care into other health and non-health settings, such as youth services, housing, probation, police, social services and local authorities. Nevertheless, uptake has proven to be low even within primary care, where there has been most research and policy interest, [13] and implementation challenging, even when significant national support, funding and delivery targets are in place, as has been shown to be the case in Scotland [16, 44]. A more in-depth understanding of implementation and delivery issues may help to explain why effects reported in efficacy studies do not always translate into effectiveness in practice [45] and which intervention elements work best in which settings for motivating and sustaining behaviour change. This is of particular relevance in light of recent evidence from large pragmatic trials that have been unable to confirm previous claims for effectiveness of IBAs in primary care and community pharmacy settings [11, 12, 30].

Overall, most delivery staff in this study felt their respective settings were appropriate for the delivery of the intervention and reported confidence in intervention delivery, despite some underlying concerns in relation to the sensitive nature of the topic. Findings concur with many of the key organisational, provider and patient level influences identified in an international review on barriers and facilitators to IBA implementation, including a lack of resources, workload, the sensitive nature of the topic and a desire not to cause embarrassment or upset [46]. In addition, our analysis highlights key barriers and facilitators to implementation common to all community settings, including delivery within public spaces, as well as those that were setting-specific, such as locating IBA delivery in proximity of alcohol sales in supermarkets. Staff from both community pharmacies and community health settings were more likely to have access to onsite support and felt more equipped in dealing with people at higher risk and signposting to alcohol services. Health and wellbeing advisors from community health settings also benefited from their existing skills and expertise in behaviour change, the provision of health advice, and knowledge of local resources and support. Other non-clinical settings in which IBA have been more recently encouraged, such as social services or probation, face challenges associated with alcohol issues being mainly seen and experienced in terms of dependency and problematic drinking [32]. This was not an issue in the settings included in this study, however, some staff felt that the success of the intervention was limited by the lack of engagement by those in higher risk categories.

Staffing issues and concerns about offending customers are commonly reported barriers to IBA implementation in community pharmacy settings [23, 24, 28]. This study helps to illustrate how these concerns can vary across and within settings and relate to beliefs about existing role boundaries and public expectations. Implementation issues relating to role legitimacy, role relevance and role support have been reported by others [32] and IBA is more likely to be accepted when perceived as compatible with existing goals and ways of working [20]. These findings provide support to existing arguments and illustrate their relevance in alternative settings. In addition, this analysis highlights how perceptions of actual or potential success are also framed in relation to existing day to day roles and responsibilities and the contexts in which they take place.

Perceived role legitimacy and relevance, alongside other organisational and leadership differences, resulted in considerable heterogeneity in both the enthusiasm for delivery and the prominence given to the intervention promotional materials both across and within different settings. Although this can be problematic in terms of intervention fidelity, it is proposed that building on the setting-specific strengths can help to facilitate a wider population reach and more extensive engagement with people at different levels of motivation to change their drinking behaviour. Bumbarger and Perkins [47] differentiate between two different types of deviations from intervention fidelity: those associated with barriers to full implementation or “drift” and those reflecting constructive adaptations initiated to better fit the implementers’ population or setting, thereby reflecting a degree of “innovation” within the overall intended intervention approach. The latter is essential for successful implementation in heterogeneous community settings, however, increased understanding of the different stages or components of behaviour change can help to identify how much flexibility is possible before the intervention ceases to be effective or defined as IBA. The content of advice and brief counselling and the skills required to result in improved outcomes is rarely evaluated in practice and this makes it difficult to draw conclusions about the mechanisms of effect and which intervention components or combinations are most effective [13].

There have been long-standing calls for more theory-informed approaches to behaviour change to support the effective design and evaluation of public health interventions and promotional campaigns. A systematic review of theoretically- informed interventions for lifestyle modification in primary care identified that those based on the TTM have been effective for smoking cessation in the long term [48]. The authors found no theoretically- informed intervention research evidence focusing on alcohol consumption in this setting. The TTM proposes that different processes are more appropriate for people in different motivational stages and that changes in decisional balance (incorporating attitude, social influence and self-efficacy) are associated with different stages of change [49]. The model of reach and engagement proposed here maps easily to intervention processes and the stages of change from the TTM [50]. Interviewees across all settings provided examples of pro-active engagement with people of varying levels of risk and motivation for behaviour change, however, their perceptions of their role relevance varied across stages. Subtle differences in emphasis were identified in relation to how delivery staff from different settings viewed their role in promoting awareness of the negative effects of drinking and motivating positive behaviour change. Different measures of intervention success may therefore be more acceptable in some settings than others. For example, increasing motivation by shifting the balance of pros and cons may be a more acceptable measure for settings more suited to focusing on earlier stages of change (pre-contemplation and contemplation), and short-term behaviour change for settings that include health professional or behaviour change roles who align more easily with later stages of change.

A recent study on exposure to revised drinking guidelines [51] based on the COM-B model [52] has demonstrated that exposure to guidelines is associated with an increase in capability (proportion who reported tracking units of alcohol consumption and considered it easier to drink safely), opportunity (proportion who perceived their lifestyle as conducive to drinking within guidelines) and reflective motivation to drink within the guidelines, and that this effect diminishes over time. Delivery of IBA within a range of community settings can help support behaviour change by increasing capability (provision of knowledge and increasing self-efficacy); opportunity (exposure to guideline information and cues to action); and motivation (increasing feedback on personal risk and desire to reduce harm).

Findings from this study suggest that public interest or engagement with IBA is likely to be influenced by a combination of perceived level of risk, motivational stage or decisional balance, and perceptions of the setting or context in which the intervention is taking place. In line with research identifying that pharmacists are viewed as “reliable advisors on health matters” [27], community pharmacy staff felt that customers were more trusting of them because of the “health care” pharmacy environment. Pharmacy staff also reported that knowing their customers helped them to approach people they felt were more at risk. Nevertheless, social desirability bias in relation to alcohol consumption may understandably be more prominent in health-related settings; the lack of familiarity with the delivery staff and non-health related setting of the supermarket or other community setting may encourage more honest responses about drinking habits. Alcohol has long been acknowledged as a sizeable component of purchases in supermarkets in the UK and routine purchasers of alcohol may be more likely to visit the supermarket more frequently than the pharmacy or be more consciousness of their alcohol buying habits or intentions when present in an environment where it is sold. This may be an area worthy of further research.

### Strengths and limitations

There are a number of limitations to this study. Heterogeneity of delivery and lack of consistency within each setting type made comparisons between accounts more complex. In the case of community pharmacies, for example, there was considerable variation in the size, layout and facilities in each premises, as well as the degree of engagement by staff. Other authors have identified that engagement with IBA may also be influenced by pharmacy type, size, location or the deprivation level of the areas in which they were based [23, 27, 30] and these elements were not evident in the accounts of our interviewees. For supermarket stores, the intervention was largely the responsibility of one individual on-site with little control over where they might be stationed and very little time to acquaint themselves with the promotional materials. The two geographically distinct community health led arms of the intervention were substantively different in terms of engagement with the programme and the research study. One was coordinated by a small team of facilitators, focused on the IBA work for the duration of the intervention and engagement and fidelity to the intervention processes was more evident. The other incorporated the intervention within existing locally commissioned health services and engagement from this arm in both the intervention and research was low. Transferability of findings may also be limited by different sampling approaches taken in different settings as well as by international differences within the chosen community settings. For example, supermarkets in the UK, although comparable to the US, differ from other European countries in the way alcohol is sold as well as their stated agendas relating to corporate social responsibility.

While most published guidance recommends that people with scores in the higher risk categories of the AUDIT-C questionnaire go on to receive the full AUDIT questionnaire [53], this would have been impractical in the settings explored in this study. Integral to the design therefore was an emphasis on being able to highlight local onward referral points of contact for each site. Finally, due to pragmatic limitations, we did not interview members of the public who had engaged or had the opportunity to engage with the intervention. Future research would benefit from incorporating these perspectives. Emphasis within the training on the importance of identifying and encouraging theory-informed fidelity to core elements of the intervention and the subsequent impact on behavior change is recommended for future work.

## Conclusions

By comparing experiences and views from delivery staff across different community settings using the same IBA intervention, findings from this study allow unique insight into the ways in which intervention acceptability and success are framed across settings, how the intervention is assimilated within everyday practice, and some of the key areas to be addressed when implementing IBAs in non-clinical community settings by staff with diverse levels of health-related knowledge, skills and support needs. The potential influence of perceived role boundaries on intervention fidelity and expectations of success, in particular, should be explicitly acknowledged and addressed.

Findings also emphasise and support the potential value of multi-setting community approaches to facilitate more inclusive engagement with IBA, complementing their use in other health-related settings. Building on the different strengths and facilitators to implementation across different types of settings and staff roles has the potential help to increase population reach and reinforce motivational shift and behaviour change by engaging members of the public at various stages of behaviour change in subtly different ways. Although essential, the need for adaptable delivery and training approaches across different setting types is, however, likely to result in methodological challenges that will need to be addressed when evaluating future interventions and setting-specific influences on behaviour change and health outcomes.

## Abbreviations

ABI: Alcohol brief intervention
AUDIT-C: Short form Alcohol Use Disorders Identification Test
IBA: Identification and brief advice
MRC: Medical Research Council
SBIRT: Screening, Brief Interventions and Referral to Treatment
TTM: Transtheoretical model

## References

[1] Rehm J, Mathers C, Popova S, Thavorncharoensap M, Teerawattananon Y, Patra J (2009). Global burden of disease and injury and economic cost attributable to alcohol use and alcohol-use disorders. Lancet.

[2] Kaner EF, Dickinson HO, Beyer F, Pienaar E, Schlesinger C, Campbell F (2009). The effectiveness of brief alcohol interventions in primary care settings: a systematic review. Drug and Alcohol Review.

[3] World Health Organization. Global strategy to reduce the harmful use of alcohol. http://www.who.int/substance_abuse/publications/global_strategy_reduce_harmful_use_alcohol/en/. Accessed 1 June 2017.

[4] HM Government. The Government's Alcohol Strategy 2012 https://assets.publishing.service.gov.uk/government/uploads/system/uploads/attachment_data/file/224075/alcohol-strategy.pdf. Accessed 1 June 2017.

[5] Kaner EF, Beyer F, Dickinson HO, Pienaar E, Campbell F. Schlesinger C, er al. Effectiveness of brief alcohol interventions in primary care populations The Cochrane Database of Systematic Reviews. 2007;2. CD004148. 10.1002/14651858.CD004148.pub3 17443541

[6] O'Donnell A, Anderson P, Newbury-Birch D, Schulte B, Schmidt C, Reimer J (2014). The impact of brief alcohol interventions in primary healthcare: a systematic review of reviews. Alcohol Alcohol.

[7] World Health Organization. WHO alcohol brief intervention training manual for primary care. World Health Organization Regional Office for Europe 2017. http://www.euro.who.int/__data/assets/pdf_file/0006/351294/Alcohol-training-manual-final-edit-LSJB-290917-new-cover.pdf?ua=1. Accessed 20 October 2018.

[8] Babor TF, McRee BG, Kassebaum PA, Grimaldi PL, Ahmed K, Bray J (2007). Screening, brief intervention, and referral to treatment (SBIRT): toward a public health approach to the management of substance abuse. Subst Abus.

[9] van Beurden I, Anderson P, Akkermans RP, Grol RP, Wensing M, Laurant MG (2012). Involvement of general practitioners in managing alcohol problems: a randomized controlled trial of a tailored improvement programme. Addiction.

[10] Butler CC, Simpson SA, Hood K, Cohen D, Pickles T, Spanou C (2013). Training practitioners to deliver opportunistic multiple behaviour change counselling in primary care: a cluster randomised trial. BMJ.

[11] Kaner E, Bland M, Cassidy P, Coulton S, Dale V, Deluca P (2013). Effectiveness of screening and brief alcohol intervention in primary care (SIPS trial): pragmatic cluster randomised controlled trial. BMJ.

[12] Heather N (2014). The efficacy-effectiveness distinction in trials of alcohol brief intervention. Addiction Science & Clinical Practice.

[13] McCambridge J, Saitz R (2017). Rethinking brief interventions for alcohol in general practice. BMJ.

[14] O'Donnell A, Kaner E (2017). Are brief alcohol interventions adequately embedded in UK primary care? A qualitative study Utilising normalisation process theory. Int J Environ Res Public Health.

[15] Coulton S, Newbury-Birch D, Cassidy P, Dale V, Deluca P, Gilvarry E (2012). Screening for alcohol use in criminal justice settings: an exploratory study. Alcohol Alcohol.

[16] Fitzgerald N, Platt L, Heywood S, McCambridge J (2015). Large-scale implementation of alcohol brief interventions in new settings in Scotland: a qualitative interview study of a national programme. BMC Public Health.

[17] Fitzgerald N, Molloy H, MacDonald F, McCambridge J (2015). Alcohol brief interventions practice following training for multidisciplinary health and social care teams: a qualitative interview study. Drug and Alcohol Review.

[18] Herring R, Thom B, Bayley M, Tchilingirian J (2016). Delivering alcohol identification and brief advice (IBA) in housing settings: a step too far or opening doors?. Drugs.

[19] Newbury-Birch D, Coulton S, Bland M, Cassidy P, Dale V, Deluca P (2014). Alcohol screening and brief interventions for offenders in the probation setting (SIPS trial): a pragmatic multicentre cluster randomized controlled trial. Alcohol Alcohol.

[20] Stead M, Parkes T, Nicoll A, Wilson S, Burgess C, Eadie D (2017). Delivery of alcohol brief interventions in community-based youth work settings: exploring feasibility and acceptability in a qualitative study. BMC Public Health.

[21] Brown TJ, Todd A, O'Malley CL, Moore HJ, Husband AK, Bambra C (2016). Community pharmacy interventions for public health priorities: a systematic review of community pharmacy-delivered smoking, alcohol and weight management interventions. BMJ Open.

[22] Todd A, Copeland A, Husband A, Kasim A, Bambra C (2015). Access all areas? An area-level analysis of accessibility to general practice and community pharmacy services in England by urbanity and social deprivation. BMJ Open.

[23] Brown S, Henderson E, Sullivan C (2014). The feasibility and acceptability of the provision of alcohol screening and brief advice in pharmacies for women accessing emergency contraception: an evaluation study. BMC Public Health.

[24] Hattingh HL, Hallett J, Tait RJ (2016). Making the invisible visible' through alcohol screening and brief intervention in community pharmacies: an Australian feasibility study. BMC Public Health.

[25] Sheridan J, Stewart J, Smart R, McCormick R (2012). Risky drinking among community pharmacy customers in New Zealand and their attitudes towards pharmacist screening and brief interventions. Drug and alcohol Review.

[26] Fitzgerald N, Youngson E, Cunningham S, Watson M, Stewart D (2015). Support for community pharmacy-based alcohol interventions: a Scottish general public survey. Public Health.

[27] Krska J, Mackridge AJ (2014). Involving the public and other stakeholders in development and evaluation of a community pharmacy alcohol screening and brief advice service. Public Health.

[28] Horsfield E, Sheridan J, Anderson C (2011). What do community pharmacists think about undertaking screening and brief interventions with problem drinkers? Results of a qualitative study in New Zealand and England. Int J Pharm Pract.

[29] Watson MC, Blenkinsopp A (2009). The feasibility of providing community pharmacy-based services for alcohol misuse: a literature review. International Journal of Pharmacy Practicee.

[30] Dhital R, Norman I, Whittlesea C, Murrells T, McCambridge J (2015). The effectiveness of brief alcohol interventions delivered by community pharmacists: randomized controlled trial. Addiction.

[31] Platt L, Melendez-Torres GJ, O'Donnell A, Bradley J, Newbury-Birch D, Kaner E (2016). How effective are brief interventions in reducing alcohol consumption: do the setting, practitioner group and content matter? Findings from a systematic review and metaregression analysis. BMJ Oen.

[32] Thom B, Herring R, Bayley M (2016). The role of training in IBA implementation beyond primary health care settings in the UK. Drugs.

[33] Health Education England. Making Every Contact Count 2018. http://makingeverycontactcount.co.uk/. Accessed 1 May 2018.

[34] Craig P, Dieppe P, Macintyre S, Michie S, Nazareth I, Petticrew M (2013). Developing and evaluating complex interventions: the new Medical Research Council guidance. Int J Nurs Stud.

[35] Drinkaware. Drinkaware https://www.drinkaware.co.uk/. Accessed 2 Feb 2018.

[36] 2CV: Middle Aged Men Campaign Development: Summary Report. 2016.

[37] Bush K, Kivlahan DR, McDonell MB, Fihn SD, Bradley KA (1998). The AUDIT alcohol consumption questions (AUDIT-C): an effective brief screening test for problem drinking. Ambulatory care quality improvement project (ACQUIP). Alcohol use disorders identification test. Arch Int Med.

[38] Saunders JB, Aasland OG, Babor TF, de la Fuente JR, Grant M (1993). Development of the alcohol use disorders identification test (AUDIT): WHO collaborative project on early detection of persons with harmful alcohol consumption--II. Addiction.

[39] Grier A, Bryant C (2005). Social marketing in public health. Ann Rev Public Health.

[40] Donovan GR, Paudyal V (2016). England's healthy living pharmacy (HLP) initiative: facilitating the engagement of pharmacy support staff in public health. RSAP.

[41] Braun V, Clarke V (2006). Using thematic analysis in psychology. Qual Res Psychol.

[42] Lincoln Y, Guba G (1985). Naturalistic Inquiry.

[43] National Institute of Health and Clinical Excellence. Alcohol-use disorders: prevention. Public health guideline PH24 https://www.nice.org.uk/Guidance/PH24. Accessed 1 May 2018.

[44] Saitz R (2014). Lost in translation: the perils of implementing alcohol brief intervention when there are gaps in evidence and its interpretation. Addiction.

[45] Heather N (2014). Interpreting null findings from trials of alcohol brief interventions. Frontiers in psychiatry.

[46] Johnson M, Jackson R, Guillaume L, Meier P, Goyder E (2011). Barriers and facilitators to implementing screening and brief intervention for alcohol misuse: a systematic review of qualitative evidence. J Public Health.

[47] Bumbarger B (2008). Perkins . After randomised trials: issues related to dissemination of evidence-based interventions. Journal of Children's Services.

[48] Bully P, Sanchez A, Zabaleta-del-Olmo E, Pombo H, Grandes G (2015). Evidence from interventions based on theoretical models for lifestyle modification (physical activity, diet, alcohol and tobacco use) in primary care settings: a systematic review. Prev Med.

[49] Schulz DN, Kremers SP, de Vries H (2012). Are the stages of change relevant for the development and implementation of a web-based tailored alcohol intervention? A cross-sectional study. BMC Public Health.

[50] Prochaska JO, Velicer WF (1997). The transtheoretical model of health behavior change. Am J Health Promot.

[51] Stevely AK, Buykx P, Brown J, Beard E, Michie S, Meier PS, Holmes J (2018). Exposure to revised drinking guidelines and 'COM-B' determinants of behaviour change: descriptive analysis of a monthly cross-sectional survey in England. BMC Public Health.

[52] Michie S, van Stralen MM, West R (2011). The behaviour change wheel: a new method for characterising and designing behaviour change interventions. Implement Sci.

[53] NHS Health Education England. e-learning for health care. An online learning resource for healthcare and socialcare professionals working to reduce alcohol related harm. 2018. https://www.e-lfh.org.uk/programmes/alcohol/. Accessed 21 Dec 2018.

